# First-in-human use of a continuous real-time atrial electrogram monitoring device after cardiac surgery

**DOI:** 10.1016/j.hrcr.2021.01.011

**Published:** 2021-01-28

**Authors:** Nitin Somasundaram, Joshua Kankam Boadu, Nicholas H. Von Bergen

**Affiliations:** University of Wisconsin – Madison, Madison, Wisconsin

**Keywords:** Atrial electrogram, Atrial epicardial wires, Cardiac surgery, Continuous real-time monitoring, Postoperative arrhythmias

## Introduction

Postoperative arrhythmias are present in 30%–60% of the more than 400,000 patients who undergo cardiac surgeries in the United States each year.^1^, ^2^, ^3^, ^4^ These arrhythmias are associated with the need for additional support, increased morbidity owing to diminished cardiac output, stroke, or, in extreme cases, additional mortality.^1^, ^2^, ^3^^,^^5^, ^6^, ^7^

Postoperative arrhythmias are most often diagnosed through analysis of electrograms displayed on the bedside monitor from surface electrodes. However, rhythm discrimination can be difficult or impossible when interpreting surface electrograms owing to the inability to distinguish the atrial and ventricular signals. In particular, on the surface electrogram, much smaller atrial signals may not be visible. Owing to this diagnostic challenge, American Heart Association (AHA) practice standards recommend using the atrial electrogram for more accurate rhythm identification.^8^ This is traditionally done using a 12-lead electrocardiogram (ECG) with the V_1_ lead attached to the atrial epicardial wire, providing both a surface ECG and an atrial electrogram on the V_1_ channel. However, this method is not in real time or continuous, limits accessibility to the electrogram, and results in missed diagnosis.

Recently, a novel device, the AtriAmp (Atrility Medical, Madison, WI), was developed to improve postoperative rhythm identification by providing continuous real-time display of the atrial electrogram on the bedside monitor. At the same time, if pacing is required, the AtriAmp can connect to a temporary pacemaker to allow atrial monitoring and pacing using a temporary pacemaker.

We describe the first-in-human use of the AtriAmp for continuous real-time monitoring of the atrial electrogram for more accurate rhythm identification in a postoperative pediatric patient.

**Figure 1:**
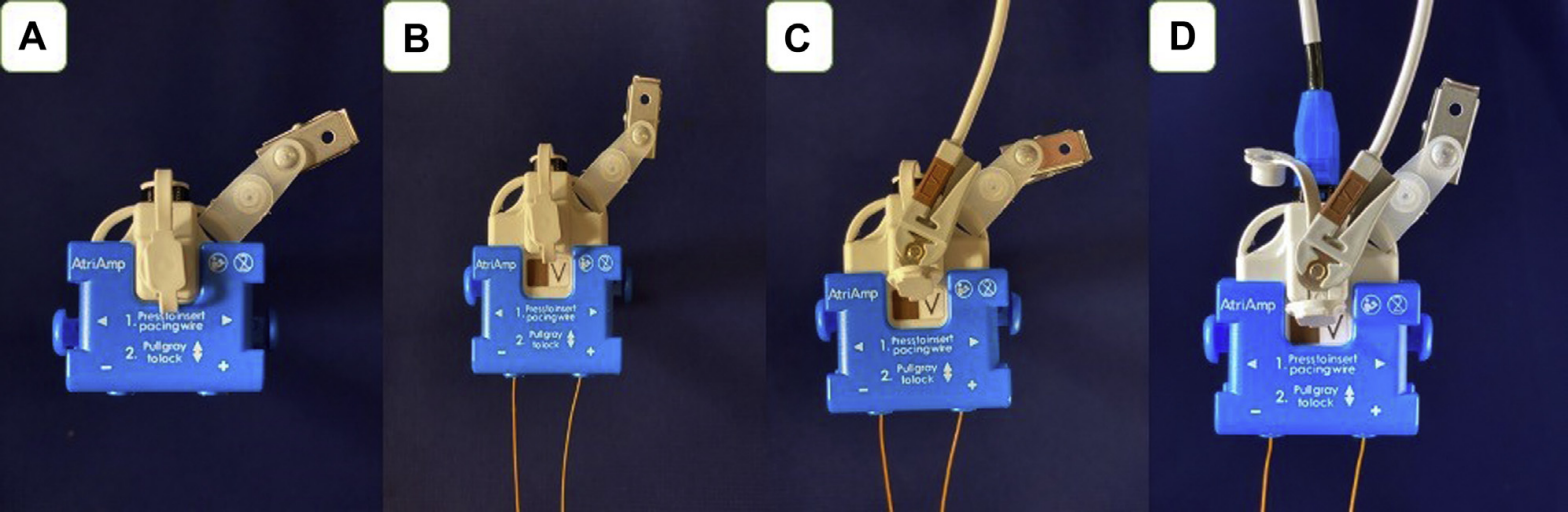
**A:** The AtriAmp (Atrility Medical, Madison, WI). **B:** The AtriAmp after connecting to the patient epicardial wires and securing the wires by sliding the gray portion into extended position. **C:** The AtriAmp after connection to both the patient epicardial wires and the V lead wire from the bedside monitor. This provides continuous atrial electrogram display on the bedside monitor. **D:** The AtriAmp connected to patient epicardial wires, the bedside monitor, and the temporary pacemaker (cable provided separately). This allows for atrial pacing through the AtriAmp using a compatible temporary pacemaker.

**Figure 2:**
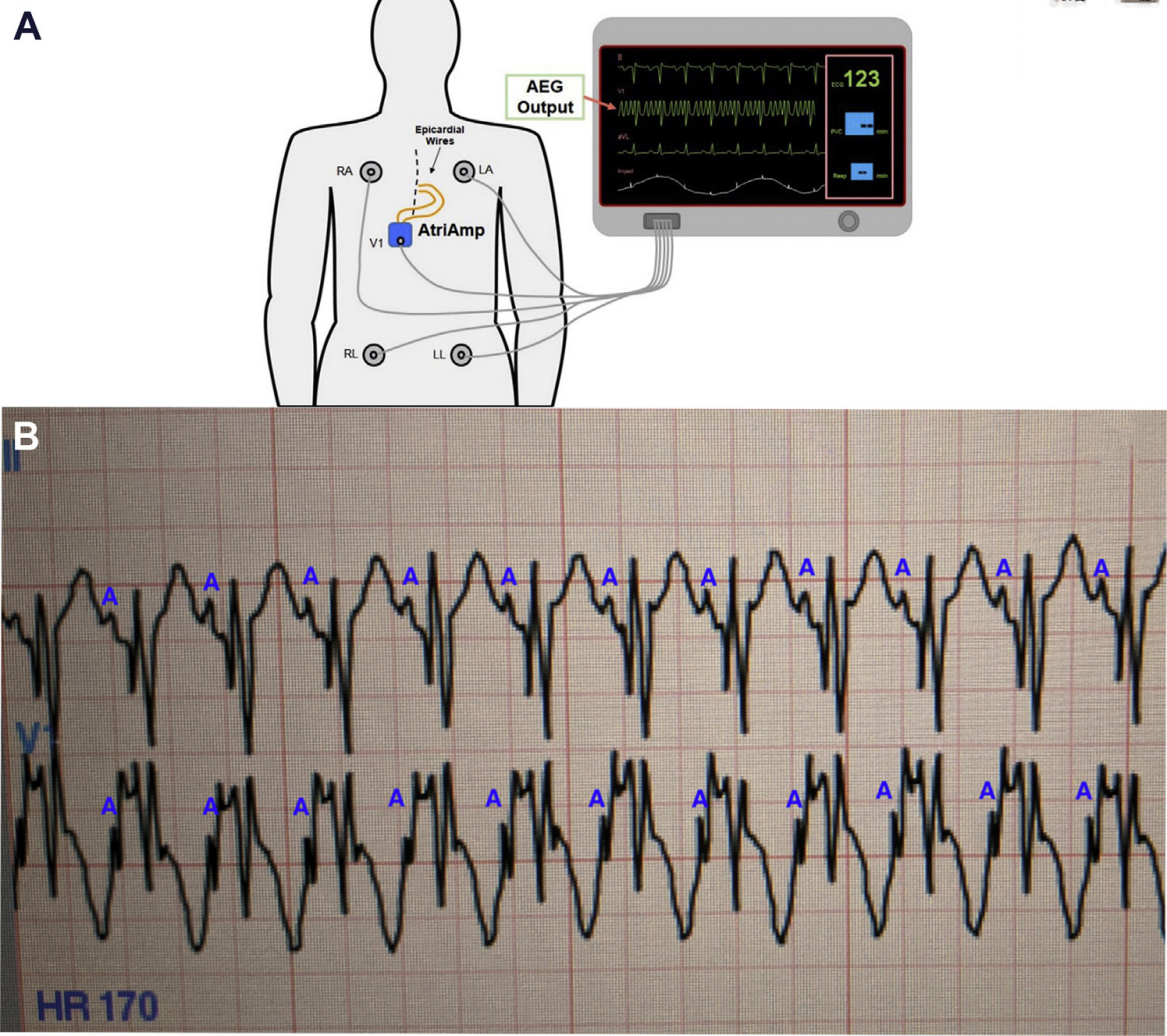
**A:** A figure depicting the attachments for continuous atrial electrogram monitoring on the bedside monitor using the AtriAmp (Atrility Medical, Madison, WI). The monitor displays the surface electrograms (limb leads) and the atrial electrogram (labeled AEG output). This electrogram is adapted for a real electrogram during an atrial arrhythmia. **B:** This patient’s surface and atrial electrogram displayed on the bedside monitor after attachment of the epicardial wires and V lead wire to the AtriAmp. The upper electrogram (labeled II) is the surface lead. The lower lead (labeled V1) is the atrial electrogram. The atrial signals on both the surface and atrial electrogram are labeled with a blue A. On the AtriAmp lead, notice the large atrial signals just prior to the QRS with an amplitude near the amplitude of the QRS signal.

The AtriAmp, a single-use device, can provide continuous atrial electrogram monitoring on the bedside monitors. To do this, the AtriAmp (Figure 1A) connects to and secures the atrial epicardial wires in the (+) and (-) AtriAmp ports (Figure 1B). The AtriAmp can then be connected to the V lead of any intensive care–type CF-certified bedside monitor on the V lead wire connection site (Figure 1C). This allows transmission of the atrial epicardial signals to the bedside monitor in real time. For optimal interpretation of the atrial electrogram, the bedside monitor should display both a limb lead channel (ie, lead I or II) and the V lead channel (the atrial electrogram). If both epicardial wires are attached directly to the atrium, the atrial epicardial signal can be evaluated by assessing the appearance of the epicardial wires in both the (+) and (-) port of the AtriAmp and the arrangement with the most prominent atrial signals can be selected. (Figure 2a and 2b).

If atrial pacing (or sensing from the pacemaker) is required, the AtriAmp may be connected to a temporary pacemaker using a separately provided cable (Figure 1D). If pacing is not required, the temporary pacemaker need not be connected. The AtriAmp is compatible with standard epicardial wires ranging from 0.84 to 2.215 mm in diameter. For temporary pacing, the AtriAmp connector cables are compatible with the Medtronic temporary pacemakers or temporary pacemakers that utilize a 2 mm shrouded connection (Oscor, Abbott, Cardiotronic).

## Case report

A term female infant was noted to have progressive fussiness and difficulty feeding over the first month of life. As the infant was from the plain clothes community, the first medical evaluation was at 1 month of age by their midwife. A murmur prompted cardiac evaluation, which discovered a large perimembranous ventricular septal defect and hypoplastic arch. Afterload reduction and diuretics were trialed but at 6 weeks of age, with continued weight loss and poor feeding, the infant presented for elective repair.

The infant weighed 2.6 kg at ventricular septal defect closure, aortic isthmus resection, and arch augmentation with an end-to-side anastomosis of the descending to ascending aorta. During the procedure, atrial and ventricular temporary epicardial pacing wires (A&E Medical, Farmingdale, NJ) were placed in a bipolar arrangement on the atrial or ventricular myocardium. The infant was transferred to the pediatric intensive care unit (ICU) after the surgery with no complications and with no residual disease.

Once in the ICU, the bedside monitor was set to display 2 electrograms, a surface lead and the V lead, to allow display of both the atrial electrogram from the AtriAmp on the V lead and the surface electrogram. The AtriAmp was the attached to patient’s atrial epicardial wires and the V lead from the bedside monitor as shown in Figure 1C. Both arrangements of the atrial epicardial wires in the (+) or (–) port of the AtriAmp were tested to determine which atrial signal was most prominent as displayed on the V lead on bedside monitor. The arrangement with the largest atrial signal was maintained (Figure 2b)

During the ICU admission, while the atrial epicardial wires were in place, the AtriAmp displayed the atrial electrogram continuously on the bedside monitor and was visible though the remote telemetry. The accurate assessment of atrial activation assisted with the identification of sinus rhythm, sinus tachycardia, and ventricular arrhythmias in this patient. The rapid confirmation of sinus rhythm through easy and accurate identification of the atrial signals was also frequently helpful.

**Figure 3:**
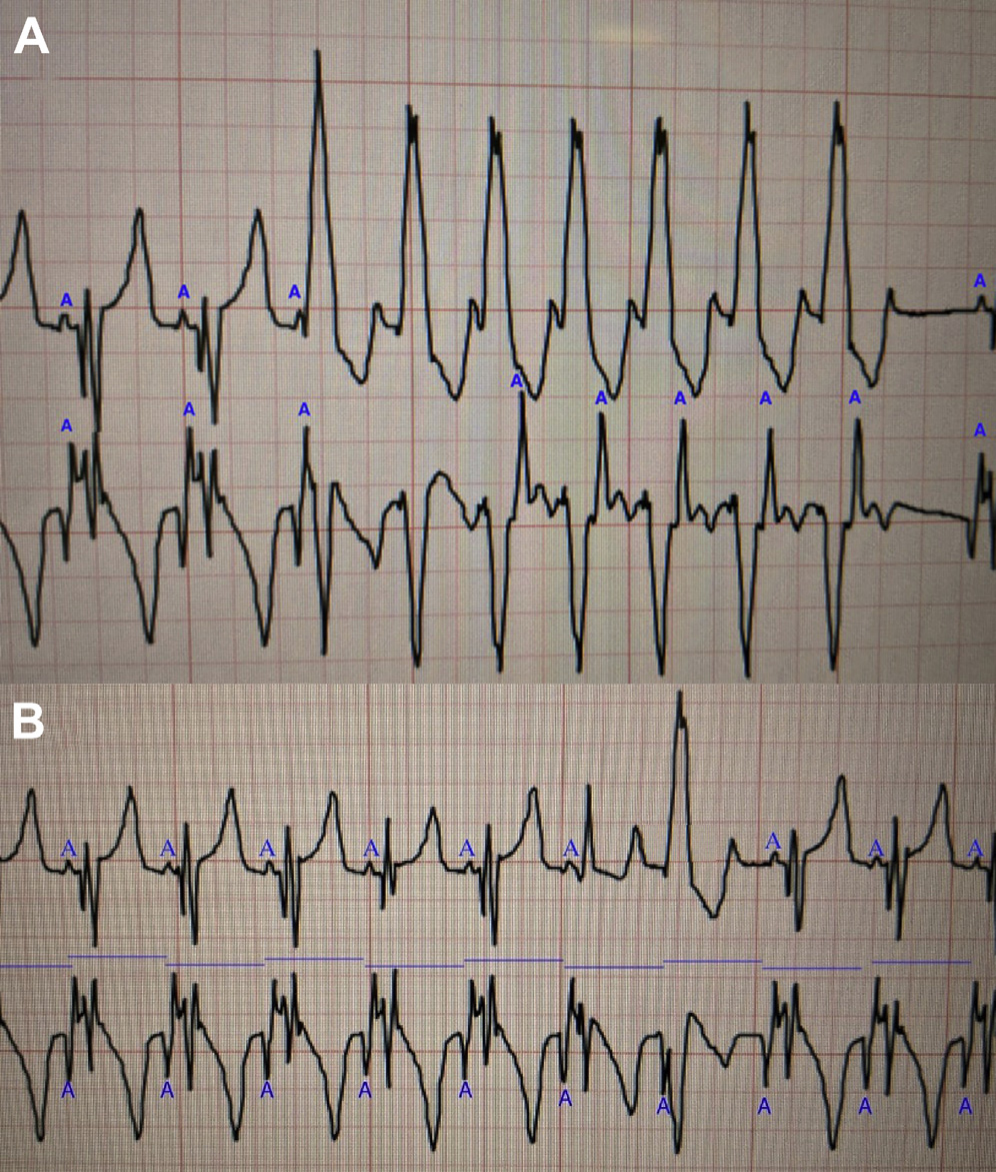
**A:** The top electrogram is lead II from the surface electrodes. The lower electrogram is the AtriAmp (Atrility Medical, Madison, WI) atrial electrogram signal. The atrial signals on the surface and on the atrial electrogram are labeled with a blue A. Note the prominent atrial signals on the atrial electrogram, which cannot be identified on the surface electrogram. The ventricular complexes increase to a rate to faster than the atrial signals, resulting in more ventricular than atrial signals. This confirms the diagnosis of a 7-beat run of ventricular tachycardia (as opposed to an atrial arrhythmia with aberrancy). The atrial signals following the wide complex ventricular beats are likely activated via retrograde conduction through the atrioventricular node. **B:** The top electrogram is lead II, and the lower electrogram is the atrial electrogram. The atrial signals are labeled with a blue A, and the blue bars all represent the same duration of time, at the rate of the atrial signals. The premature ventricular contraction (PVC) was easily diagnosed when confirming that there was no proceeding atrial signal to suggest a premature atrial complex with aberrancy. The atrial electrogram also showed no change in the atrial to atrial (A-A) intervals. This, with an early wide complex ventricular signal, confirms the diagnosis of a ventricular arrhythmia. In this case, there was a fusion beat (the fifth from the right), and a PVC.

The patient demonstrated occasional premature ventricular complexes and a few short runs of a wide complex rhythm. Overnight, using the surface leads, an episode of wide complex rhythm was tentatively diagnosed as either a supraventricular tachycardia with aberrancy or a ventricular arrhythmia. Review of the atrial electrogram confirmed a 7-beat run of ventricular tachycardia, as demonstrated in Figure 3a. As shown, the ventricular signals accelerate ahead of the atrial signals, proving the ventricular origin of the arrhythmia. The AtriAmp signal allowed easy identification of the atrial signals, which were not visible on the surface electrogram, making the diagnosis.

Premature ventricular complexes (PVCs) were also noted and could be confidently diagnosed with the simple and accurate identification of the atrial signals (Figure 3b).

The identification of the ventricular arrhythmia, including the PVCs, resulted in additional diligence with monitoring, but ultimately required no intervention. The arrhythmias decreased and subsided by discharge. The epicardial wires were removed on postoperative day 2. The patient continued routine postprocedure convalescence, though she continued to have slow weight gain and marginal feeding. She was discharged home on postoperative day 10.

## Discussion

We describe the first-in-human use of the AtriAmp, a device that can allow continuous and real-time atrial electrogram monitoring using the bedside monitoring equipment. This device is the first device to provide continuous atrial electrogram monitoring while, if required, at the same time allow for atrial pacing using a temporary pacemaker.

Atrial electrogram monitoring has been used for more accurate rhythm identification for the last 40 years and is even recommended in the American Heart Association Practice Standards for postoperative ECG monitoring.^8^^,^^9^ However, despite a long history and strong recommendation for its use, the atrial electrogram remains underutilized. We believe that this is in large part owing to the lack of technology to provide real-time and continuous atrial electrogram monitoring in the ICU. Most current technologies require an ECG technician and the modification of a 12-lead ECG for an atrial electrogram. This requires both time and expertise, resulting in delayed or missed diagnosis. The AtriAmp overcomes these hurdles by providing the highest-quality signal in real time on the bedside monitor. This also allows providers to take advantage of the remote monitoring/telemetry capabilities and allows retrospective evaluation of the arrhythmias through the bedside telemetry system.

Though this case report describes the first-in-human use of the AtriAmp in a single patient with ventricular arrhythmias, we have noted that the atrial electrogram has been especially useful for the confirmation of sinus tachycardia and the identification of junctional rhythms in subsequent patients. Given this, we expect this to be especially helpful for early identification and more accurate diagnoses of arrhythmias in the pediatric population in particular. At the time of this manuscript, the AtriAmp has not been used in the adult population, though we expect it will be especially helpful for rapid and accurate atrial fibrillation diagnosis.

## Conclusion

We describe the first-in-human use of the AtriAmp, which allows continuous postoperative atrial electrogram monitoring after cardiac surgery. The improved ability to identify the atrial electrogram resulted in more accurate diagnosis in this pediatric patient.

**Key Teaching Points**

• Postoperative arrhythmias are common after cardiac surgery. These arrhythmias may increase morbidity and mortality and lengthen hospital stay.
• Current technology, using surface electrograms, is inadequate for accurate arrhythmia identification and diagnoses.
• The atrial electrogram allows for greater accuracy than surface electrograms and is recommended for rhythm identification by the American Heart Association.
• This case report describes the first-in-human usage of the AtriAmp, a device to allow continuous real-time display of the atrial electrogram from atrial epicardial wires after heart surgery.
